# Ergonomic Analysis of Dental Work in Different Oral Quadrants: A Motion Capture Preliminary Study among Endodontists

**DOI:** 10.3390/bioengineering11040400

**Published:** 2024-04-19

**Authors:** Sophie Feige, Fabian Holzgreve, Laura Fraeulin, Christian Maurer-Grubinger, Werner Betz, Christina Erbe, Albert Nienhaus, David A. Groneberg, Daniela Ohlendorf

**Affiliations:** 1 Institute of Occupational Medicine, Social Medicine and Environmental Medicine, Goethe University Frankfurt, Theodor-Stern-Kai 7, 60590 Frankfurt am Main, Germany; 2 Institute of Dentistry, Department of Dental Radiology, Goethe University Frankfurt, Theodor-Stern-Kai 7, 60590 Frankfurt am Main, Germany; 3 Department of Orthodontics, University Medical Center of the Johannes Gutenberg-University, Augustusplatz 2, 55131 Mainz, Germany; 4 Principles of Prevention and Rehabilitation Department (GPR), Institute for Statutory Accident Insurance and Prevention in the Health and Welfare Services (BGW), 22089 Hamburg, Germany

**Keywords:** dentists, muscular skeletal disorders, working posture, ergonomic risk, quadrants, kinematic analysis

## Abstract

Background: Dentists, including endodontists, frequently experience musculoskeletal disorders due to unfavourable working postures. Several measures are known to reduce the ergonomic risk; however, there are still gaps in the research, particularly in relation to dental work in the different oral regions (Quadrants 1–4). Methods: In this study (of a pilot character), a total of 15 dentists (8 male and 7 female) specialising in endodontics were measured while performing root canal treatments on a phantom head. These measurements took place in a laboratory setting using an inertial motion capture system. A slightly modified Rapid Upper Limb Assessment (RULA) coding system was employed for the analysis of kinematic data. The significance level was set at *p* = 0.05. Results: The ergonomic risk for the entire body was higher in the fourth quadrant than in the first quadrant for 80% of the endodontists and higher than in the second quadrant for 87%. For 87% of the endodontists, the ergonomic risk for the right side of the body was significantly higher in the fourth quadrant compared to the first and second quadrant. The right arm was stressed more in the lower jaw than in the upper jaw, and the neck also showed a greater ergonomic risk in the fourth quadrant compared to the first quadrant. Conclusion: In summary, both the total RULA score and scores for the right- and lefthand sides of the body ranged between 5 and 6 out of a possible 7 points. Considering this considerable burden, heightened attention, especially to the fourth quadrant with a significantly higher ergonomic risk compared to Quadrants 1 and 2, may be warranted.

## 1. Introduction

Current systematic reviews and meta-analyses confirm the high prevalence of musculoskeletal disorders (MSDs) among dentists [1,2,3,4,5]. According to Soo et al., the 12-month prevalence varies between 68 and 100% [4]. The most stressed parts of a dentist’s body are the neck, lower back and shoulders [1,2,3,4,5]. According to Kumar et al. [6], endodontists showed the highest MSD prevalence compared to dentists with other specialisations, which led to a high 12-month prevalence of MSDs (61–89%) [7,8]. This high percentage is partly due to adopting unfavourable working postures without sufficient breaks and long working hours in static positions [3,9,10]. Static posture at work is cited as the main risk factor for MSDs [11]. In occupational science, a static working posture is defined as a posture that is maintained for longer than four seconds with little or no fluctuation around a fixed force level that is maintained by muscles and other body structures [12,13]. These working postures are often found among endodontists [6,14]. Considering the day-to-day work of an endodontist, this may involve very long treatments on one tooth and an unfavourable static posture [15]. This is the result of so-called forced postures that occur when a body posture is dictated by the work process over a long period of time without sufficient compensatory movements being possible [16]. The risk of MSDs is already increased if work is carried out in the same position for longer than 40 min [17], such as in the case of endodontists, where the working area is limited to the root canal system in the tooth and the posture cannot vary to a great extent. The length of treatment time during root canal treatment clearly correlates with the strain on the neck [18]. Furthermore, symptoms of MSDs increase with the number of years of practice [19,20,21,22,23]. It is difficult, or even impossible, to take breaks during root canal treatment because the patient’s mouth should be held open for the entire session using a rubber dam. Preventive measures for MSDs include daily stretching exercises [7,11], the use of modern and ergonomic instruments [11,24], ergonomic dental chairs [22], magnifying loupes [24,25] or prism glasses and ergonomic training [26]. The SOPEZ project (“Study for the optimization of ergonomics in the dental practice—musculoskeletal disorders in dentists and dental assistants”) [27] was launched in order to obtain more information about MSDs and the possible influencing factors, including those in the various dental specialities, so that suitable preventive measures can be recommended. This study forms part of the SOPEZ project [27] in the context of which various questions have already been published [28,29,30,31].

Current studies [30,32,33] indicate that the ergonomic risk for dentists working in the different regions of a patient’s mouth (Q1–Q4) varies, although either not all four quadrants were considered [32,33] and/or different regions within the quadrants were worked on [30,32]. Overall, the research situation is insufficient in this respect.

This research gap is, therefore, the subject of this present analysis, which has a pilot study character. A comparison of the same dental activity between the four quadrants has not yet been carried out in the analysis of the development of MSDs in dentists. In addition, we consequently aim to analyse whether the ergonomic risk differs within the four dental quadrants in one typical activity for endodontists (root canal preparation). The dental activity was recorded by continuous kinematic data using an inertial motion capture system [34]. Furthermore, an ergonomic assessment of this dental treatment by endodontists was carried out using the Rapid Upper Limb Assessment (RULA) [35,36,37], a globally used, validated assessment method for the ergonomic evaluation of workplaces associated with WRULDs (work-related upper limb disorders) [35]. The German Society for Occupational Medicine and Environmental Medicine (DGAUM) and the Society for Occupational Science (GfA) recommend the RULA method in their S1 guideline “Physical strain on the back due to load handling and forced postures in the work process” to assess strain on the shoulders and upper back [38].

Since the RULA procedure is originally an observational procedure that assesses a momentary observation using the paper–pencil method, multiple modifications [37,39] were made to ensure that this procedure for assessing the upper limb or trunk provides all the necessary information for answering our research objective. For example, the observer who decides which work task is to be assessed [40] and which joint angles are predominantly used was replaced by inertial motion capture (IMC) [37].

In this way, exact quantitative, continuous kinematic data [39], including the joint angular velocity, are available for the entire work process in each joint. This allows a risk assessment of the entire body (only total scores are possible in the original version) as well as for individual body parts on both sides of the body in isolation (right/left) or in combination at any time during the work process. Based on the angular velocity, it is possible to differentiate the movement according to the static or dynamic movement components, and this also allows pauses to be identified. After these modifications, RULA was used as a superordinate systematic structure (scoring table and structure) in order to compare the results with other studies. The implementation of these biomechanical components allowed the entire movement sequence to be analysed and not just a single frame as a maximum value. RULA was, therefore, the assessment of choice due to the large number of steps already available for calculating the final score, on the one hand, and the focus on the upper body on the other, since dental activities are predominantly performed in a sitting position. These modifications [37,39] led to other risk assessment tools [40], such as the Quick Exposure Check (QEC), the Ergonomic Workplace Analysis method developed by the Finnish Institute of Occupational Health (FIOH) [40], the American Conference of Governmental Industrial Hygienists Hand Activity Level (HAL) threshold limit values method, Job Strain Index (JSI), Rapid Entire Body Assessment (REBA) and EN 1005-3 standard, not being selected as the assessment tool of choice for determining ergonomic risk. The latter two tools must be excluded, for example, as this study is intended to compare dental work in the different quadrants where the focus is on the strain on the entire upper body, in particular on the neck, trunk, shoulders, arms and wrists (RULA) (sedentary work of dentists) and is, thus, not focused on the leg work (REBA) or the high exertion of force (EN 1005-3 standard). The Occupational Risk Assessment (OCRA) could also be excluded since the aim of this study was to record quantitative data and was not a subjective assessment of the employee. In addition, the integration of kinematic data into the RULA evaluation method used here was tested against the traditional application of RULA using observers in a comparative study [31]. 

Assuming that there might be different levels of stress during treatment in the individual quadrants, this could also be taken into account when scheduling appointments. In addition, the practitioner should be able to pay more attention to an ergonomic posture in a quadrant with a higher overall risk of MSDs. 

## 2. Materials and Methods 

### 2.1. Subjects

In this study, a total of 15 dentists (8 male/7 female) specialising in endodontic treatment were measured while performing root canal treatment on a dummy head. Their average age was 32.7 ± 4.3 years. The dentists were supported by their dental assistants so that they could work as realistically as possible. Men and women aged 18–65 years who were right-handed were included. The dentists were also asked to wear magnifying glasses since the use of optical magnification aids is particularly common in root canal treatments (due to the extremely small working area). In this way, the treatment situation was modelled as realistically as possible. Table 1 shows an overview of the study’s participants.

Exclusion criteria comprised current injuries to the musculoskeletal system (e.g., herniated discs, injuries to the spine), rheumatic diseases, severe restrictive malformations (scoliosis) of the spine or stiffening of the spinal joints (pathological or surgical), genetically determined muscle diseases and surgical interventions that occurred less than 2 years previously [27].

The study was approved by the ethics committee of the Faculty of Medicine at the Goethe University Frankfurt am Main (356/17).

### 2.2. Measuring System

Body posture was recorded throughout the treatment using the inertial motion capture (IMC) system “Xsens MVN” (Enschede, The Netherlands). This is a kinematic full-body inertial measurement system that records synchronised video data as well as joint angles, centre of mass and factory-made calibrated sensor data. The sampling rate was 240 Hz [34]. IMU (inertial measurement unit) -based systems exhibit accuracy levels that are notably contingent upon the specific characteristics of the plane under evaluation [41]. While the sagittal and frontal planes exhibit a satisfactory agreement of 2°, the transverse plane presents a slightly wider margin of approximately 5° [41]. It is notable that these values closely correspond with the established tolerances, typically manifesting within the range of +/−1°.

In order to achieve the best results, the manufacturer recommends carrying out the recordings in the “no level” mode. Each subject was measured in a seated position with the pelvis remaining at a specified height.

**Figure 1:**
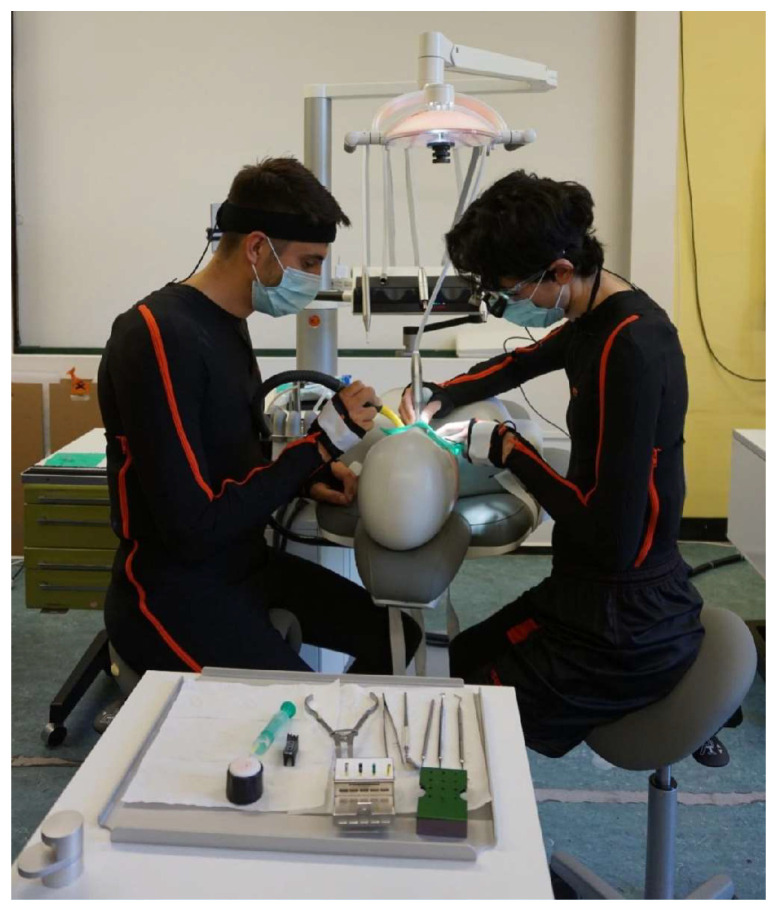
Dentist and dental assistant in the Xsens suit during treatment on the dummy head in basic concept 3.

The subjects were dressed in matching Lycra suits (sizes S–XXL) as well as a headband, gloves and foot pads, as shown in Figure 1. Seventeen sensors (motion trackers), a bodypack and the battery were attached to the suit using Velcro fasteners at strategically favourable points and were connected to each other via cables. The anatomical points for the placement of the sensors are specified by the manufacturer. Exact placement is not necessary. The subjects were able to move freely. The motion tracker (MTx) is a complete miniature inertial measurement unit with integrated gyroscopes, accelerometers, magnetometers and a barometer.

The data were transmitted wirelessly from the bodypack to a so-called access point that was connected to the PC via a cable. Before the data were recorded, the subjects were calibrated. Only when the calibration quality “good” was displayed could the recording be started. After the recordings were complete, the data were subjected to so-called “HD reprocessing” and subsequently analysed [34].

### 2.3. Experimental Setup and Realisation

**Figure 2:**
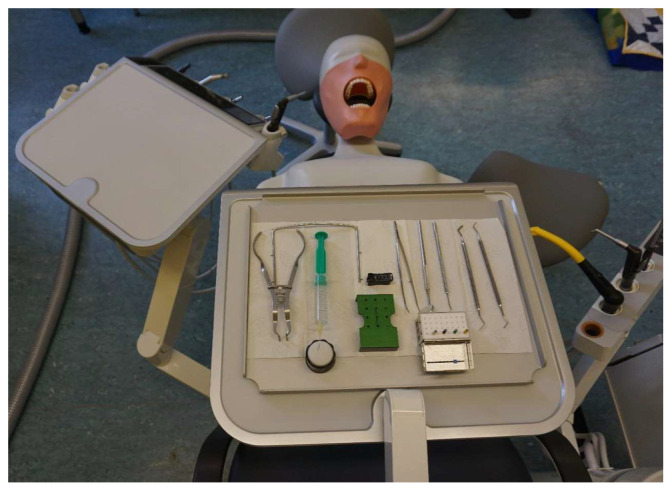
Dummy head attached to the treatment centre with the prepared tray according to basic concept 4.

The subjects worked not only in basic concept 1, which is predominant in Germany, but also in four different basic concepts [42,43,44,45] using a saddle chair. In order to map these and to create a comparable work situation, the measurements were carried out in a laboratory environment at the Institute for Occupational Medicine at the Goethe University in Frankfurt am Main. The study participants performed a standardised task typical for endodontists on a dummy head (see Figure 2).

To compare the ergonomic risk between the individual quadrants (Q1–Q4), the same activity was performed in each quadrant on the sixth tooth (i.e., tooth numbers 16, 26, 36 and 46). This procedure was repeated for each concept.

Root canal preparation with the following substeps or tasks was carried out in each case:

Task 1 An already prepared rubber dam was positioned by the dentist. The dental assistant provided assistance.
Task 2 Trepanation of the tooth was undertaken so that all canals could be probed with an ISO 20 K-file [46].
Task 3 Root canal preparation was undertaken with hand files (K-files, ISO 35-45 [46]) using circumferential filing with a specified working length (WL); adjustment of the WL was made by the dental assistant.

Rinsing with H_2_O and dry blowing was employed between the use of the individual files. 

When finished, rinsing and blowing dry was undertaken.

Task 4 The rubber dam was removed by the dentist. The dental assistant provided assistance.

After written consent was given for the performance and collection of personal data, calibration of the Xsens MVN for each participant, using a neutral posture and short walking sequence, was obtained. Subsequently, the measurements were commenced in basic concept 1, followed by concepts 4, 2 and, finally, 3.

**Figure 3:**
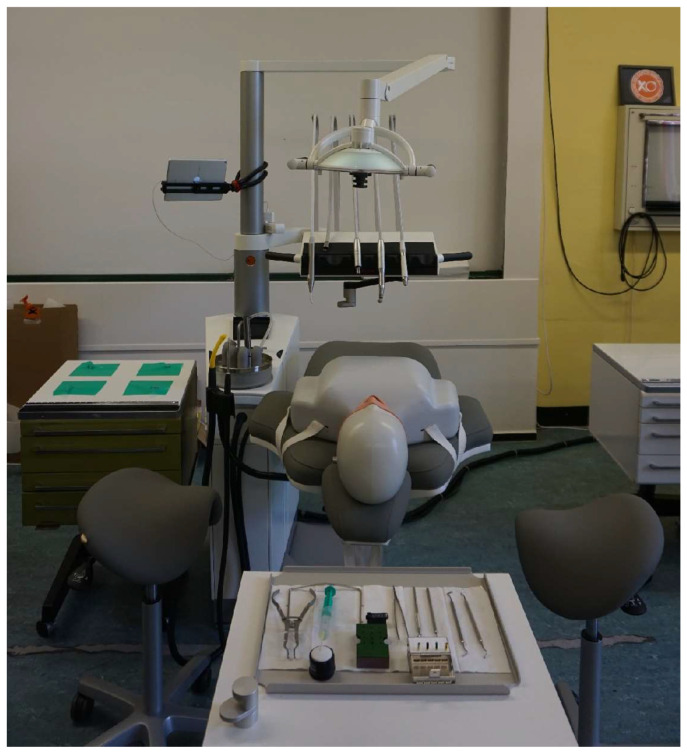
Standardised starting position before the measurements in basic concept 3 (swivel unit) with the prepared tray and dental lamp aligned vertically above the dummy head.

The tray was always prepared exactly as shown in Figure 2 and Figure 3 before commencing each concept. 

Before the start of each quadrant, the dental lamp was positioned vertically above the dummy head in a standardised starting position (Figure 3). During the treatment, the test subjects were permitted to adjust the light themselves. In contrast, the position of the dummy head was only allowed to be changed before the measurement and, thus, adapted to the respective quadrant (as patients would normally perform this movement themselves). 

The treatment chair for basic concepts 1–3 was always in a more horizontal position (patient lying or sitting), whereas, in concept 4, the patient was always positioned to be completely flat [45].

In order to visualise the start and end of the measurement, the subjects were instructed to assume a start/end position before and after each individual task. The left and right hands were placed on the respective thighs, and their face and gaze were directed towards the dummy head.

The entire measurement was filmed in parallel with an iPad Air (3rd generation) in order to trace any deviations and assign the respective movements. The measurements and camera recordings were synchronised with MVN Analyze 2019.2.0 from Movella Inc. (Enschede, The Netherlands).

### 2.4. Rapid Upper Limb Assessment (RULA)

McAtamney et al. [35] originally developed RULA in 1993 to record musculoskeletal stress in workplaces where work-related diseases of the upper extremities are common (for RULA worksheet see Figure S1 in Supplementary Materials). No complicated equipment is required for this assessment. A statement can be made quickly about the posture, muscle function and external load that people experience. In addition, a scoring system provides an overview of the level of strain on the individual body parts.

In order to integrate RULA into the objective and continuous data of the IMC system, all RULA limits had to be defined quantitatively. For this purpose, the RULA protocol was adapted according to Table 2. The integration of inertial data into RULA has been published and applied in this study [39].

As the IMU-calculated angles are the actual measured joint angles, parallaxes, which can occur in classic 2D images or videos, can be excluded. In addition, the static and dynamic points of Steps 6 and 13 are particularly relevant modifications of the classical RULA template to include muscle work. 

### 2.5. Data Processing

The data were analysed using MATLAB^®^ vR2018a software (The MathWorks Inc., Natick, MA, USA). 

The slightly modified RULA coding system, as defined in Table 2, was used. The following target values per quadrant were derived from the resulting RULA scores in relation to both the total RULA score and the individual, body-regional RULA scores for each subject in order to enable a differentiated assessment of the ergonomic risk:

1. RULA score:

The median and the interquartile range (IQR) were calculated from all RULA values for the individual quadrants, i.e., one value per quadrant;

2. Relative average risk score over time (Rel. av. RST):

The median and IQR of Rel. av. RST provide information about the relative time spent in the respective RULA score (scores 1–7). This renders it possible to scrutinise the ergonomic risk in the different quadrants.

The following formula was used for this:

Relative time spent in RULA score 1 × 1 + relative time spent in RULA score 2 × 2 + relative time spent in RULA score 3 × 3 (...) + relative time spent in RULA score 7 × 7

The higher the RULA score and the higher the Rel. av. RST, the higher the ergonomic risk.

The following body parts were examined in the individual quadrants:

1. RULA total score (“Final overall”): this includes the body parts from the right or left with the greater risk in each case;
2. RULA—total score on the right (“Final overall right”): this looks at the right half of the body (Step 1 right, Step 2 right, Step 3 + 4 right, Step 9, Step 10);
3. RULA—total score on the left (“Final overall left”): this considers the left half of the body (Step 1 left, Step 2 left, Step 3 + 4 left, Step 9, Step 10);
4. Local scores:

-Upper Arm Score (left and right)**-**RULA Step 1-Lower Arm Score (left and right)-RULA Step 2-Wrist Score (left and right)-RULA Steps 3 + 4-Neck Score-RULA Step 9-Trunk Score-RULA Step 10

### 2.6. Statistical Analysis

The socio-demographic data of the study participants were checked for normal distribution using the Shapiro–Wilk test. As these were found to be normally distributed, the mean value, the standard deviation and the tolerance range were calculated. 

The Kolmogorov–Smirnov–Lilliefors test did not show a normal distribution for the kinematic data; therefore, non-parametric tests, such as the Friedman test, with post hoc tests were used and followed by multiple Conover comparisons with Bonferroni–Holm correction to calculate differences between the individual quadrants. The significance level was set at *p* < 0.05.

## 3. Results

Table 3 shows the results of the RULA analysis. The medians of the corresponding RULA scores and the relative average risk scores over time (Rel. av. RST) were calculated for each quadrant. 

Figure 4, Figure 5, Figure 6, Figure 7, Figure 8, Figure 9, Figure 10, Figure 11, Figure 12, Figure 13 and Figure 14 show the significant differences between all four quadrants determined by the Conover–Iman comparisons.

### 3.1. RULA Score

In general, the RULA total score (“Final overall”) as well as the RULA total score for the right (“Final overall right”) and left side of the body (“Final overall left”) contained all RULA values between 5 and 6 out of a total of 7 achievable points. This means that the ergonomic risk is so great that further measures or ergonomic changes should be implemented immediately [35].

The maximum achievable score varied for the remaining body parts with values of 3–6. Therefore, the risk cannot be reliably assessed as described above.

**Figure 4:**
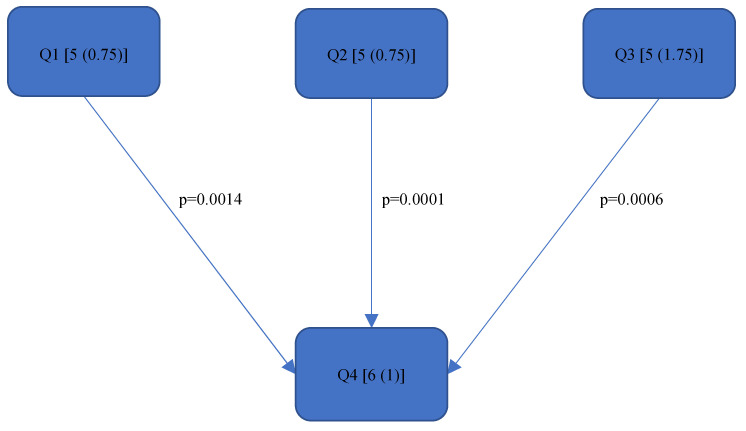
RULA score “Final overall”.

**Figure 5:**
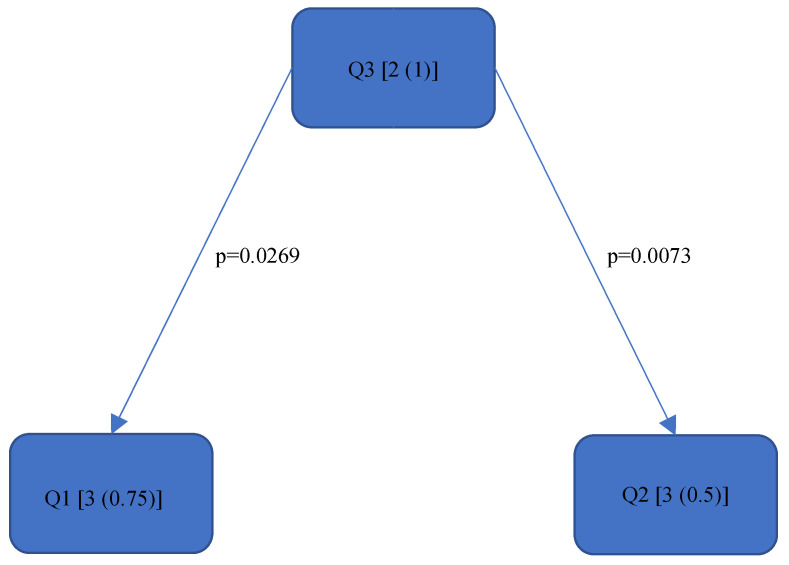
RULA score “Left Lower Arm”—Step 2.

**Figure 6:**
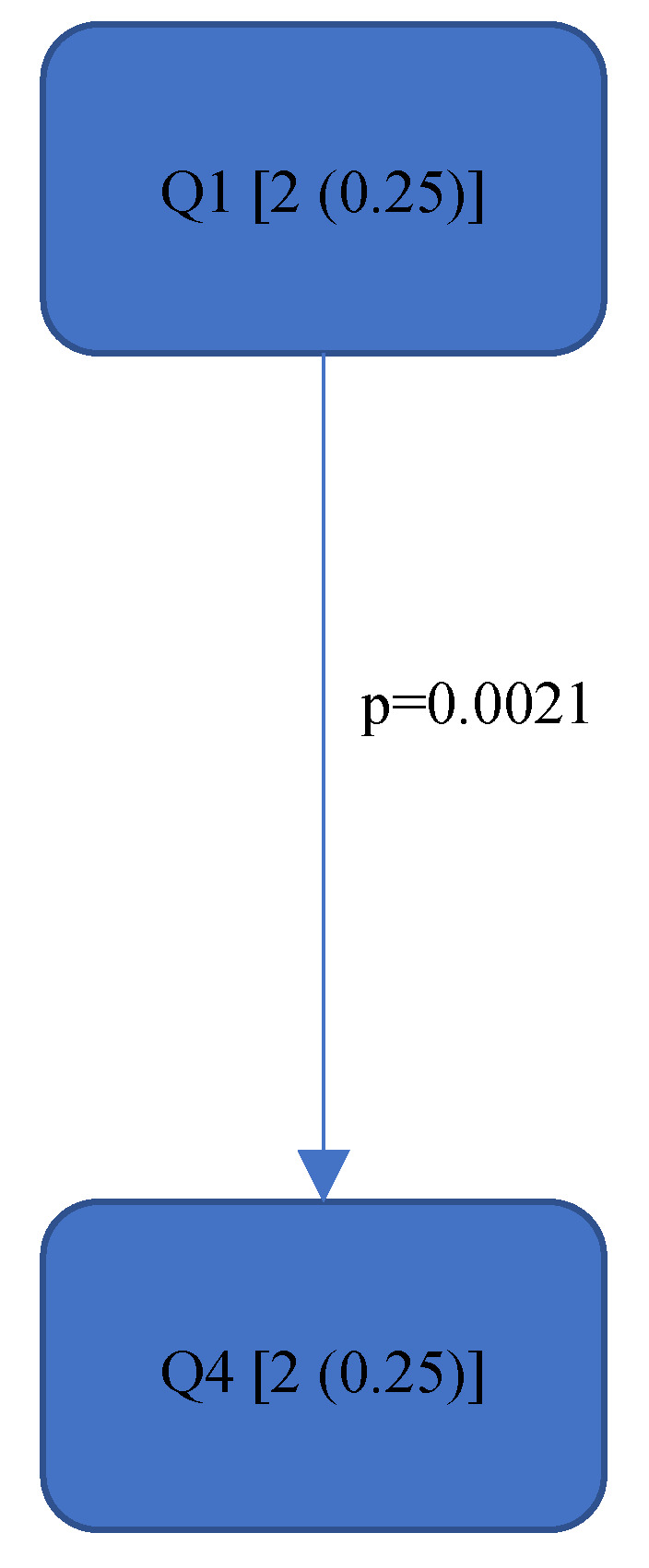
RULA score “Right Upper Arm”—Step 1.

**Figure 7:**
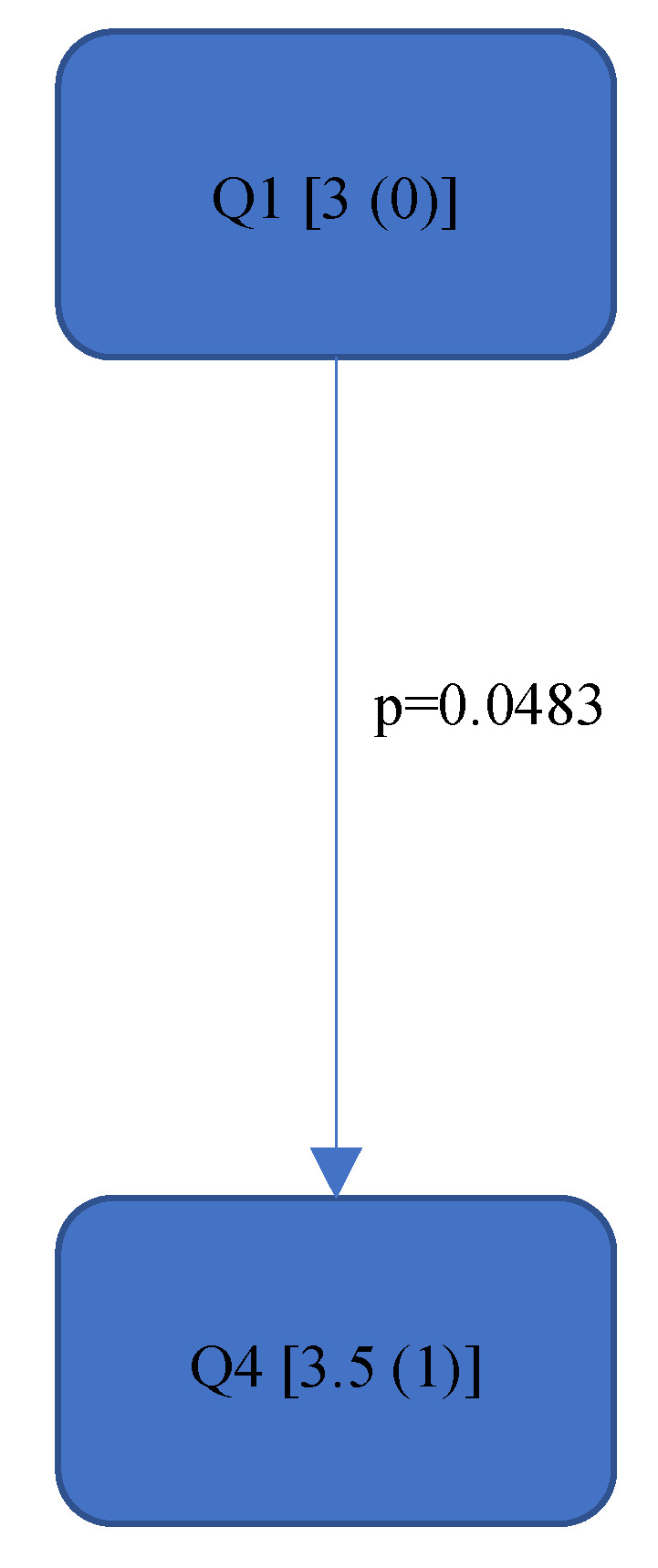
RULA score “Neck”—Step 9.

However, significant differences (*p* < 0.05) were found between the individual quadrants in the “Final overall” score as well as in the left forearm, right upper arm and neck. These are shown in Figure 4, Figure 5, Figure 6 and Figure 7 according to the individual local scores with the corresponding *p*-values.

In the “Final overall” score, Quadrant 4 showed the greatest load with a median of 6 (1) compared to the other quadrants (see Figure 4). The right upper arm and neck were responsible for this score, each showing a significantly higher risk in the fourth quadrant than in the first quadrant. 

There were no significant differences between the individual quadrants in the “Final overall right and left”, left upper arm, right forearm, wrists and trunk scores.

Figure 4, Figure 5, Figure 6 and Figure 7:

Quadrants 1–4 (Q1-Q4) are shown with their RULA scores (median and IQR) in square brackets; significant differences are illustrated by arrows, with their corresponding *p*-values (Bonferroni–Holm corrected), that lead from the ergonomically lower to the higher risk quadrant.

### 3.2. Relative Average Risk Score over Time (Rel. av. RST)

**Figure 8:**
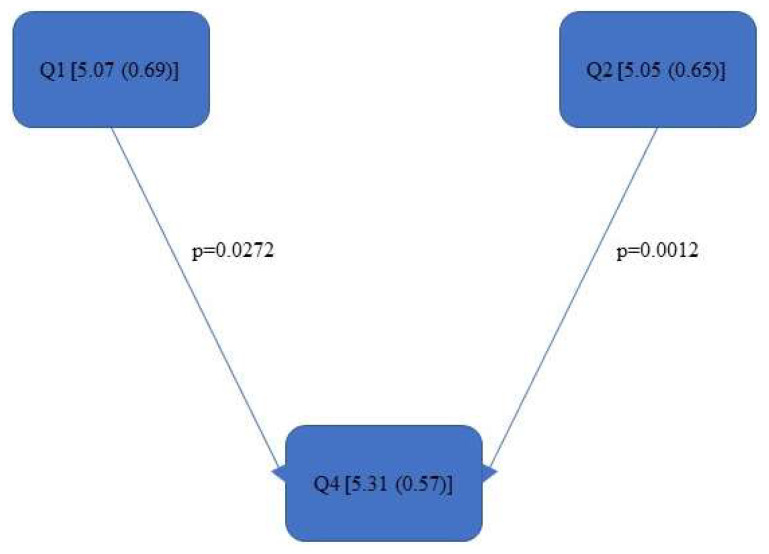
Rel. av. RST “Final overall”.

**Figure 9:**
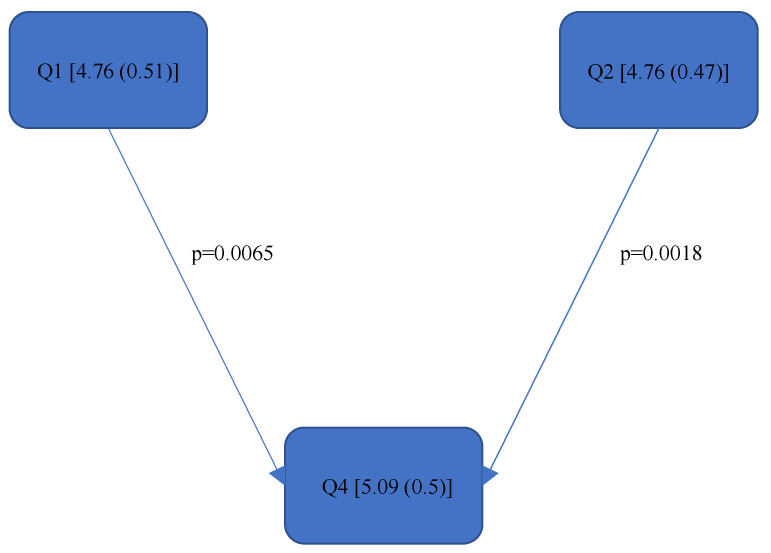
Rel. av. RST “Final overall right”.

**Figure 10:**
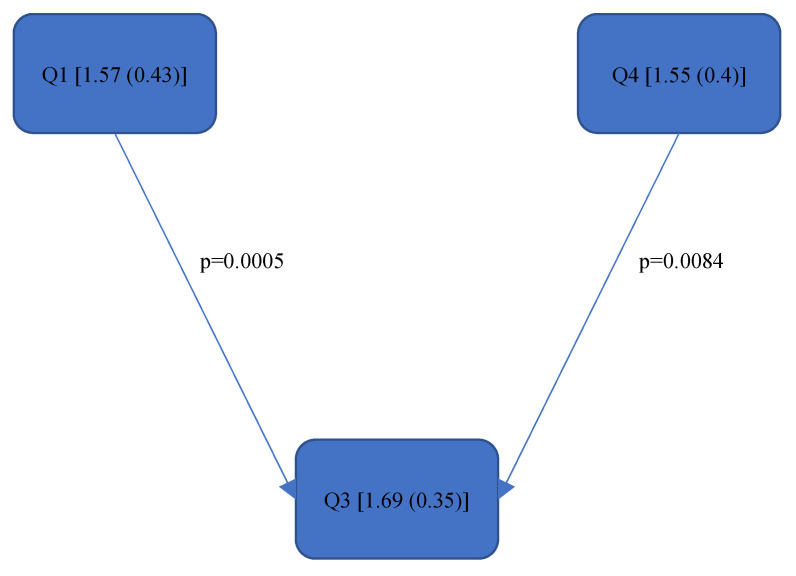
Rel. av. RST “Left Upper Arm”—Step 1.

**Figure 11:**
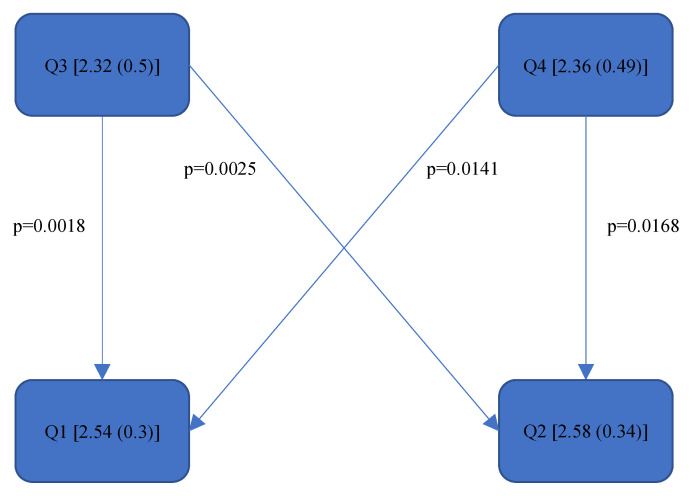
Rel. av. RST “Left Lower Arm”—Step 2.

**Figure 12:**
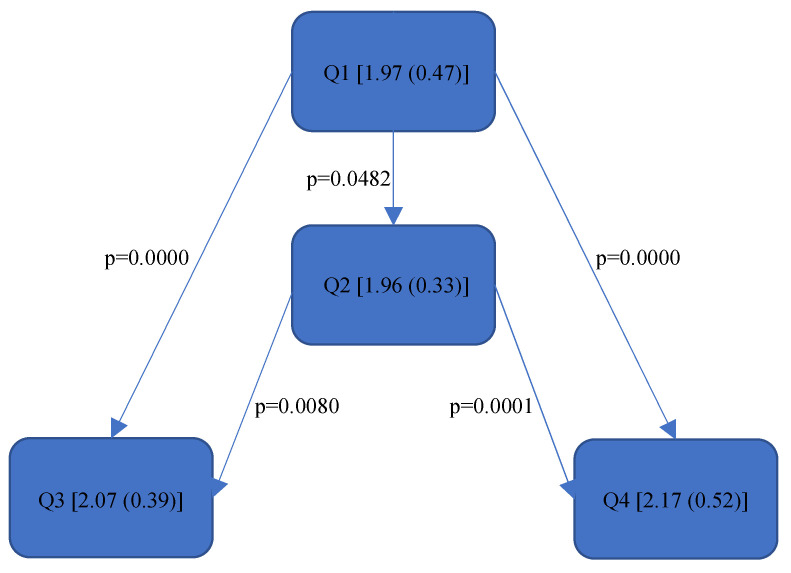
Rel. av. RST “Right Upper Arm”—Step 1.

**Figure 13:**
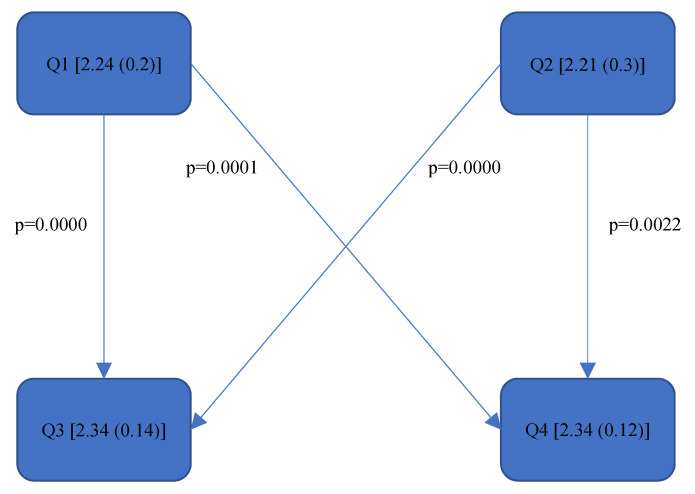
Rel. av. RST “Right Lower Arm”—Step 2.

**Figure 14:**
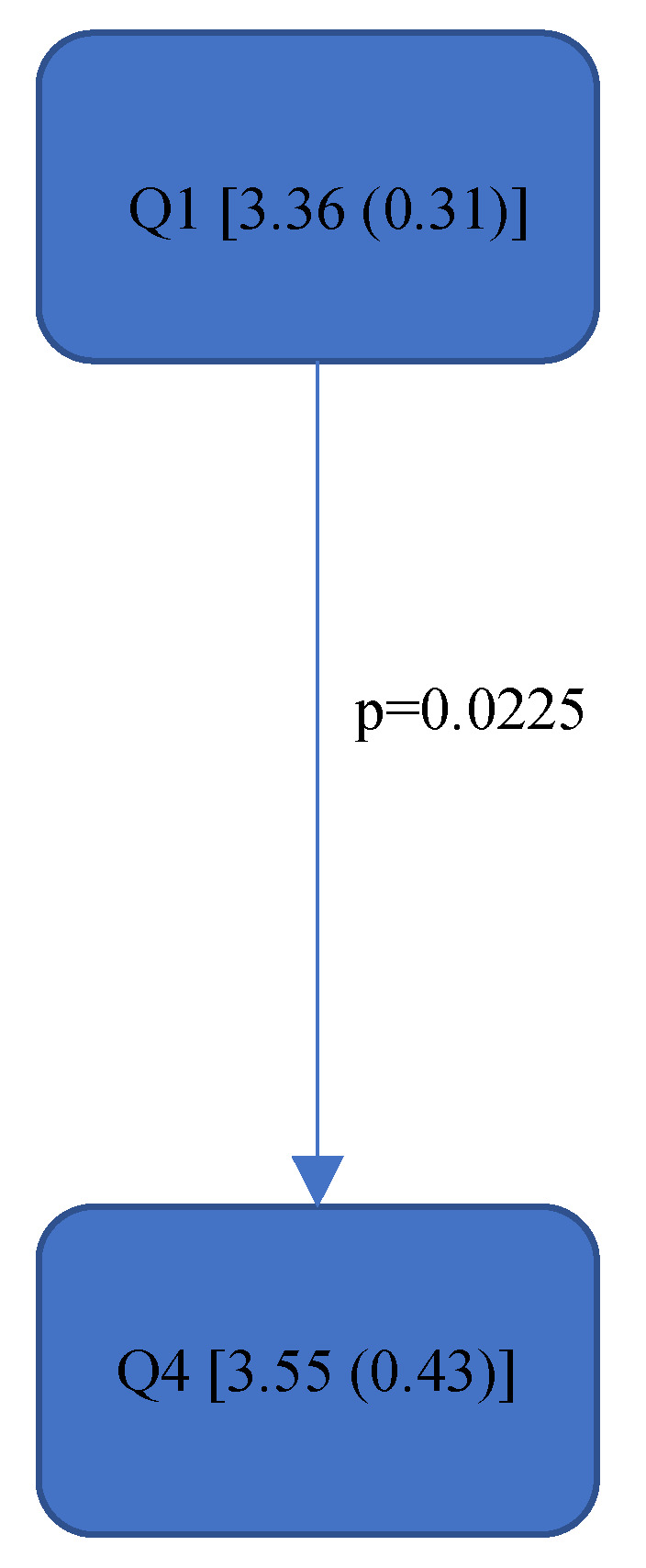
Rel. av. RST “Neck”—Step 9.

As this value takes into account the relative time spent in the respective RULA score, it reacts somewhat more sensitively than the RULA value. The differences are shown in Figure 8, Figure 9, Figure 10, Figure 11, Figure 12, Figure 13 and Figure 14.

In the “Final overall”, the Rel. av. RST showed a significantly higher load in the fourth quadrant with a value of 5.31 (0.57) than in Quadrants 1 and 2 (5.05 (0.65)—5.07 (0.69)). The numerical value of Quadrant 3 (5.05 (0.75)) was also lower here than in Quadrant 4 (5.31 (0.57)) but without significance. The fourth quadrant was determined to be ergonomically riskier than Quadrant 1 and Quadrant 2. This was also reflected in the right side of the body (“Final overall right”). The ergonomic risk was higher in the fourth quadrant than in Quadrants 1 and 2. 

Since there were no significant differences in the “Final overall left”, a body part on the right side must be responsible for the fact that Quadrant 4 performed worse in relation to the upper jaw. This was also noticeable in the right upper arm. The subjects experienced the least strain in Quadrant 1, followed by Quadrant 2. The greatest strain was experienced in Quadrants 3 and 4, although there were no significant differences between them.

The right forearm was also subjected to significantly less stress in Quadrants 1 and 2 than in the other quadrants. Therefore, the right arm is exposed to a greater ergonomic risk when working in the lower jaw than in the upper jaw. 

The neck was exposed to a greater risk when working in Quadrant 4 than in Quadrant 1. For the left upper arm, Quadrants 1 and 4 were better than Quadrant 3. The work in Quadrants 1 and 2 placed more strain on the left forearm than in Quadrants 3 and 4; this is in direct contrast to the right forearm. However, the differences in the left arm were not reflected in the “Final overall left” score. No differences in the quadrants were found in either the wrist or trunk scores.

All in all, it can be said that averaged over the treatment concepts and tasks, the right arm and the neck in the fourth quadrant were subjected to so much strain that this was reflected in the overall physical posture. 

Figure 8, Figure 9, Figure 10, Figure 11, Figure 12, Figure 13 and Figure 14:

Quadrants 1–4 (Q1-Q4) with Rel. av. RST (median and IQR) are shown in square brackets; significant differences are illustrated with arrows, with their corresponding *p*-values (Bonferroni–Holm corrected), that lead from the ergonomically lower to the higher risk quadrant.

## 4. Discussion

In general, the values of the RULA total score “Final overall” as well as for the “Final overall right” and “Final overall left” were 5 out of a total of 7 achievable points. The “Final overall” in the fourth quadrant reached a value of 6. According to McAtamney et al. [35], further measures or ergonomic changes should be immediately implemented in order to minimise the risk of MSDs. The fact that the prevalence of MSDs among dentists, in general [1,2,4,5], and endodontists [6,7,8,14], in particular, is high has already been demonstrated several times in previous surveys. Thus, the newly available kinematic data from the present study can now provide indications of the ergonomic causes. However, it should be noted that this is a preliminary study; therefore, further analyses should be carried out on the basis of these results. Furthermore, since compared to other dental specialisations, an endodontist predominantly treats one tooth for a considerable length of time in a mostly unfavourable static posture [15], the primary aim of this study was to determine whether there is a difference in the ergonomic risk of endodontists between the individual quadrants, and, if so, which parts of the body are subjected to different loads. With regard to the quadrant comparison, the results of the RULA score are only partially consistent with those of the Rel. av. RST. For example, according to Figure 4, there are significantly better scores for the RULA score in the “Final overall” for Quadrants 1 (5 (0.75)), 2 (5 (0.75)) and 3 (5 (1.75)) than for Quadrant 4 (6 (1)), while in the Rel. av. RST “Final overall” (Figure 8), only Quadrants 1 (5.07 (0.69)) and 2 (5.05 (0.65)) were significantly better than Quadrant 4 (5.31 (0.57)). It should be noted that the Rel. av. RST provides more information about the distribution of the data. The median and IQR (here, this concerns the RULA score) of two different data series can, for example, be completely identical, i.e., if the median and IQR are the same, the percentage of the individual RULA values within a quadrant can also be different; this scenario is taken into account in the Rel. av. RST (see the calculation of the Rel. av. RST under “*Data processing*”). The Rel. av. RST is, therefore, more accurate. Accordingly, it can be said that endodontological activity in Quadrant 4 is riskier than in Quadrant 1 and Quadrant 2. This contrasts with the results of Weitbrecht et al. [30], where, among other things, the ergonomic risk for oral surgeons in the first quadrant was found to be significantly greater than in the other quadrants. As the analysis of oral surgeons is also part of the SOPEZ project [27], as is the present study, Weitbrecht’s work has a comparable study design. However, the oral surgeons carried out their work in the individual quadrants in different regions: in the maxilla, a displaced canine was removed from the palate, while, in the mandible, a wisdom tooth extraction was performed in the posterior region. Therefore, it cannot be determined with certainty whether the treatment in the different quadrants was solely responsible for the different ergonomic risks or whether the different regions within a quadrant (the canine region and the posterior region) were the decisive factors. Furthermore, the differing results could be due to the different working methods of the oral surgeons and endodontists. Oral surgeons work almost exclusively under direct vision because the left hand is not free to hold a mirror as it is occupied by an instrument for holding and protecting the neighbouring soft tissue structures. This requires the practitioner to bend their upper body and neck quite excessively, especially when working in the upper jaw area. In contrast, endodontists are increasingly working under indirect vision since this has been proven to contribute to a better overall ergonomic posture [25,33,47,48].

Kamal et al. [32] also found ergonomic differences between the positions in the mouth for dental students working on a dummy head. It was found to be ergonomically more favourable to work palatally with indirect vision in the maxillary anterior region than in the mandibular posterior region on the left with predominantly direct vision. However, their study did not examine all four quadrants as in the present study; the treatment involved different regions (the maxillary anterior region and mandibular posterior region), while the ergonomic assessment was purely visual and was not based on kinematic data. In addition, the students were observed from different angles by two specialists for 60 s during their activities on a dummy head and assessed using the so-called DEA rubric. In the study by Corrales Zúniga et al. [33], the ergonomic posture of fourth-year dental students was also rated better in the maxilla than in the mandible, even though a tooth was treated in the maxilla that was one position further back than in the mandible. This result is similar to the results of the present study. However, not all four quadrants were compared with each other in Corrales Zúniga’s study; only the same activity was performed in the left second upper molar and the right first lower molar on a dummy head, and these were analysed geometrically and visually. 

In order to be able to make clear statements about the influence of treatment in the different quadrants on ergonomics, further studies would be desirable, particularly with regard to the categorisation of Quadrant 3.

Based on the more meaningful Rel. av. RST, several conclusions can be drawn regarding the ergonomic risk of the individual body parts. The right upper and lower arm are subjected to greater strain in the lower jaw than in the upper jaw. The dentists tended to sit more towards the 9.00 o’clock position when working on the lower jaw as opposed to the upper jaw (more towards the 11.00 o’clock position). In order to gain a direct view of the lower jaw, the upper body must be turned strongly to the left; thus, the right shoulder is raised more, and the right elbow is moved further away from the body. The longer this static posture is held, and the further away the arm is from the body, the higher the joint movement occurs and the less ergonomic the position becomes. The neck was subjected to less strain in the first quadrant than in the fourth quadrant since, here, the positioning of the dentist (the four-handed technique) presumably required the greatest neck flexion, rotation and tilting. The significant, additional load on the left forearm in the upper jaw in contrast to the lower jaw was probably due to the different height of the forearm; the forearm had to be raised more for working in the upper jaw than in the lower jaw, the angle was then steeper (corresponding to the joint angle > 100° [39]), and the ergonomic risk increased. The left upper arm was exposed in Quadrant 3 (Rel. av. RST: 1.69 (0.35)) to a higher ergonomic risk than in Quadrant 1 (Rel. av. RST: 1.57 (0.43)) and Quadrant 4 (Rel. av. RST: 1.55 (0.4)). The exposure in Quadrant 2 (Rel. av. RST: 1.6 (0.54)) did not differ significantly from the other quadrants; however, it can be assumed from the numerical value that the left upper arm tends to be less strained when treating the right side of a patient than when treating the left side. The left hand predominantly holds the mirror to examine the root canals under indirect vision and is not used to hold the cheek or tongue (as in other specialities of dentistry). Quadrants 1 and 4 are closer to the practitioner’s upper body than Quadrants 2 and 3. Due to the law of leverage, more static holding work is required in the left upper arm when treating the left side of the patient in order to hold the mirror in position. No significant differences were found in the wrists or trunk during treatment in the different quadrants.

As part of the SOPEZ project [27], Holzgreve et al. [29] compared, amongst other things, the load level of the individual body parts among the endodontists measured here by determining the ergonomic risk. This was lowest in both upper arms. This was followed by the trunk, the neck, the wrists and, finally, the forearms which were exposed to the greatest ergonomic risk. However, the different quadrants were not taken into account. In order to draw conclusions regarding the strength training of individual body parts, further studies should take into account the level of strain on the individual body parts. Furthermore, these findings could be helpful for dentists with existing MSDs.

According to Weitbrecht et al. [30], who carried out kinematic posture analyses in oral surgeons in a comparable study setup, some body parts did not even reach the so-called “risk threshold”, i.e., working in the different quadrants does not necessarily have a notable and possibly damaging influence on the individual body parts, even if there is a significant difference. Therefore, we conclude that the same ergonomic risk does not necessarily exist for the body parts that were subjected to significantly more stress in individual quadrants, especially in the fourth. Here, it is necessary to remind oneself of the greater general ergonomic risk for endodontists in the fourth quadrant and that the level of stress per working day in forced postures depends primarily on the cumulative duration of the static postures [23,49]. In addition to the ergonomic measures already investigated, such as the magnification devices [4,25,26,50,51], indirect vision [25,48], the type of dental chair [26,51], dental instruments [11,25,26], ergonomics training [25,26], regular physical activity [4,11,17,52,53,54], strength training [55], stretching exercises [25] and breaks [52,53], care should be taken when making appointments to ensure that dental activities in the fourth quadrant are well spread out over the week and that not too many are scheduled in one day. Endodontists generally work in accordance with an appointment system, so it is perfectly feasible for an endodontist not to carry out root canal treatments one after the other in the fourth quadrant but, rather, to spread them out over the working week. Nevertheless, endodontists working in the fourth quadrant should still pay more attention to adopting the correct ergonomic posture.

Concept 4 should be mentioned, in particular, in which the practitioner does not treat mainly at 9.00 o’clock, as in basic concepts 1–3, but at the 12.00 o’clock position with mostly indirect vision; this is in contrast to concepts 1–3 (mainly direct vision). According to Roll et al. [25], increased indirect vision should contribute to a reduction in MSDs. Therefore, it would be interesting to determine whether training in indirect vision has a positive effect on the measurement data. As the subjects were used to working in basic concept 1, which is predominant here in Germany, and most of them had not worked in any other concept before, the results may have been different if the test subjects were equally familiar with all four concepts or if the subjects were only measured in their familiar concept. Another limiting factor is that the analysis took place in vitro, and the results were to be tested in vivo. In addition, the practitioners were not able to choose their own treatment chair according to their individual seating comfort; only a saddle chair was provided that could only be adjusted in height. It also needs to be clarified if there is a difference between wearing different types of magnifying glasses or even using a microscope, which is often used by endodontists. Although the study investigated which parts of the body were subjected to more or less stress in a particular quadrant, the different basic stresses of the individual parts of the body in relation to the maximum stress were not taken into account. 

With regard to the reliability of the RULA method for assessing ergonomic risk, the literature is inconclusive [56,57,58,59]. The RULA method showed reliable results and a more significant correlation to MSDs in comparison with other representative observation methods (OWAS, REBA, LUBA and NERPA) [58,59]. It is increasingly criticised that the ergonomic risk assessment by RULA depends on the subjective expert assessment of the observers [56]. We were able to take these criticisms into account with our modifications. This source of error should be avoided by direct measurement using inertial sensors [39]. In addition, a high-resolution and continuous recording of the workload over the entire course of time is made possible; this scenario is not possible when using the traditional application using observers.

As already mentioned in the introduction, the RULA method including our modifications, therefore, served as a superordinate systematic structure (scoring table and structure) for a better comparison of the data with other studies. Not only was the “classic” RULA method digitised, but the entire course of movement was recorded and used for the assessment. In this way, many limitations of the RULA method [37,56,58] can be eliminated by modifying and implementing the assessment tool in kinematic data. Thus, analogous to the original RULA overall score, an initial assessment can now be made; in addition, each joint angle or joint angular velocity of the entire movement can be analysed in detail and in isolation. Since RULA also takes into account the load on the entire body with a focus on the upper body, and dental activities are predominantly performed in a sitting position, RULA was the assessment of choice [40]. In this context, it should be noted that, in line with Chiasson [40], there is no best assessment tool, only the most suitable method for the respective issue/workplace.

The accuracy of the combination of RULA with IMU sensors was validated by the work of Huang et al. [60], although deviations between the original paper–pen RULA method, which dominates in the scientific field, and the combination of the observational method (RULA) and inertially collected data have also been reported [31]. However, since in many areas, the paper–pen RULA values deviate systematically from the RULA values generated with inertially collected data [31], these paper–pen RULA values are less decisive for comparison in the different quadrants but, rather, can be used for the general assessment of ergonomic risk and are, therefore, only a secondary subject of this study. Nevertheless, this deviation should be taken into account when categorising the ergonomic risk according to McAtamney et al. [35] when using the RULA values of the “Final overall”, “Final overall right” and “Final overall left”. 

In future investigations, the different activities/tasks of endodontists and their influence on ergonomics should be analysed, as well as the influence of the different regions (anterior region/posterior region) within the quadrants or the extent to which the results correspond with the results obtained for dental assistants. The height ratio between the dentist and dental assistant may also have had an influence on the measurement data since the patient chair had to be adjusted either to the height of the dentist or the assistant or to a height between the two. The chair height, in particular, is associated with incorrect posture of the upper limbs and can lead to MSDs [61]. 

## 5. Conclusions

The present study of the ergonomic risk in the individual quadrants in the dental work of endodontists has shown that this varies both in the overall posture and for certain parts of the body. The ergonomic risk is higher in the fourth quadrant than in Quadrants 1 and 2. This also applies to the right side of the body. The right upper and lower arm are subjected to more strain in the lower jaw than in the upper jaw. From an ergonomic point of view, working in the fourth quadrant of a patient’s body is considerably less favourable for the neck than in the first quadrant. The left upper arm is subjected to more strain in the third quadrant than in Quadrants 1 and 4. The left lower arm is exposed to a greater ergonomic risk in the upper jaw than in the lower jaw. In addition, the ergonomic risk of endodontists is generally high, with Quadrant 4 performing the worst, so that the practitioner may be able to adapt to these ergonomic conditions individually. However, since the study situation with regard to the ergonomic risk of the quadrants is still inadequate, further research should be conducted in this area in the future. It would also be interesting to know by how much the load is greater in the fourth quadrant compared to the other quadrants and what influence the different regions (anterior regionposterior region) have within the quadrants. Scheduling where the fourth quadrant does not have to be treated cumulatively would be difficult to implement in everyday treatment, especially as patients in pain or unexpected cases can also burden the endodontists. To prevent musculoskeletal disorders, but especially in the case of existing complaints, endodontists could draw on this knowledge and focus more explicitly on their own ergonomics. The participants were not yet able to draw on this ergonomic knowledge, meaning that the results could look different with the newly acquired knowledge, including in everyday treatment.

## Figures and Tables

**Table 1:** Socio-demographic data of the study participants with average values, standard deviations and tolerance range.

Parameter	Dentist	Dental Assistant
Gender		
Male	8	1
Female	7	14
Age (years)	32.7 ± 4.3 [22.91; 42.42]	27.4 ± 6.1 [13.33; 41.33]
Height (cm)	176.6 ± 8.9 [156.23; 196.97]	170.8 ± 6.7 [155.36; 186.24]
Weight (kg)	72.1 ± 11.9 [44.88; 99.25]	69.3 ± 8.4 [49.65; 88.85]

**Table 2:** Specific adjustment parameters to calculate the RULA score. We refer to the steps as defined by McAtamney et al. [35].

Worksheet Steps	Parameters	Modifications of the RULA Parameters
Step 1	Raise shoulder Abduction of the upper arm Resting/leaning on the forearm	The IMC system calculates the elevation of the shoulder girdle; if the angle was >5°, then +1 was added to the “Upper Arm Position” [39]. For an angle > 45°, +1 was added to the “Upper Arm Position” [37]. As there is generally no support for the arms during dental work, the value was set to 0 across the board [37].
Step 3	Lateral hand bend	For a radial deviation > 10° or an ulnar deviation < −10°, +1 was added to the “Wrist Score” [37].
Step 4	Turning the forearm or hand	For twists in the neutral range between 45° and −45°, +1 was added to the “Wrist Twist Score”; for twists in the final range of movement between 90° and 45° or between −45° and −90°, +2 was added [39].
Step 6	Muscle use with “Wrist & Arm Score”	For static or repetitive muscle work, +1 was added. Static muscle work: calculation of the intervals based on the angular velocities (ω) of the shoulder joint that define static/dynamic movements. The static movement started when ω < 5°/s and ended when ω ≥ 10°/s or the angular difference was ≥ 7.5° and duration > 10 s. The sagittal and frontal movements of the shoulder joint were taken into account. In addition, no support of the arm was permitted; the centre of the wrist had to be above L5. Repetitive muscle work: calculation of the mean power frequency (MPF) taking into account the wrist movement (extension/flexion) and forearm rotation (wrist and elbow rotation). For the MPF of a joint > 0.5 Hz, +1 was added.
Step 7 + 14	Force or load for “Wrist & Arm Score” (Step 7) and “Neck, Trunk, Leg Score” (Step 14)	Since all dental instruments weigh less than 2 kg, the value was set to 0 across the board.
Step 9	Neck rotation and neck tilt to the side (frontal plane)	For a rotation or inclination > 10° or <−10°, a value of +1 was added to the “Neck Score” [37].
Step 10	Upper body rotation and upper body tilt (frontal plane)	For a rotation or inclination > 10° or <−10°, a value of +1 was added to the “Trunk Score” [37].
Step 11	Leg position	As the test subject’s legs and feet were permanently supported and balanced by their activity, a value of +1 was awarded across the board.
Step 13	Muscle use for “Neck, Trunk, Leg Score”	For static or repetitive muscle work, +1 was added. Static muscle work: analogous to “Step 6”, on the basis of angular velocities (ω) of the neck/cervical spine and lower back/lumbar spine with the condition that all three degrees of freedom of the cervical spine or lumbar spine had to be <5°/s. Repetitive muscle work: analogous to “Step 6”, taking into account all three degrees of freedom of the cervical spine and lumbar spine. For an MPF > 0.5 Hz of a joint, in one of the three degrees of freedom, +1 was added.

**Table 3:** Median RULA values and the median Rel. av. RST values in all four quadrants for the individual body parts.

Body Part (Maximum RULA Score)	Quadrant 1		Quadrant 2		Quadrant 3		Quadrant 4	
	RULA Score (IQR)	Rel. av. RST (IQR)	RULA Score (IQR)	Rel. av. RST (IQR)	RULA Score (IQR)	Rel. av. RST (IQR)	RULA Score (IQR)	Rel. av. RST (IQR)
Final overall (7)	5 (0.75)	5.07 (0.69)	5 (0.75)	5.05 (0.65)	5 (1.75)	5.05 (0.75)	6 (1)	5.31 (0.57)
Final overall right (7)	5 (1)	4.76 (0.51)	5 (1)	4.76 (0.47)	5 (1.25)	4.84 (0.61)	5 (1)	5.09 (0.50)
Final overall left (7)	5 (1)	4.86 (0.78)	5 (1)	4.83 (0.76)	5 (1)	4.84 (0.80)	5 (1.50)	5.06 (0.56)
Left Upper Arm—Step 1 (6)	1 (1)	1.57 (0.43)	1.50 (1)	1,60 (0.54)	2 (1)	1.69 (0.35)	2 (1)	1.55 (0.40)
Left Lower Arm—Step 2 (3)	3 (0.75)	2.54 (0.30)	3 (0.50)	2.58 (0.34)	2 (1)	2.32 (0.50)	2 (1)	2.36 (0.49)
Left Wrist—Steps 3 + 4 (6)	4 (1)	4.23 (0.44)	4 (0.50)	4.27 (0.26)	4 (0)	4.25 (0.25)	4.5 (1)	4.45 (0.30)
Right Upper Arm—Step 1 (6)	2 (0.25)	1.97 (0.47)	2 (0)	1.96 (0.33)	2 (0)	2.07 (0.39)	2 (0.25)	2.17 (0.52)
Right Lower Arm—Step 2 (3)	2 (0)	2.24 (0.20)	2 (0)	2.21 (0.30)	2 (0)	2.34 (0.14)	2 (0)	2.34 (0.12)
Right Wrist—Steps 3 + 4 (6)	4 (0.25)	4.09 (0.42)	4 (0)	4.14 (0.17)	4 (0)	4.21 (0.32)	4 (0)	4.02 (0.38)
Neck—Step 9 (6)	3 (0)	3.36 (0.31)	3 (0.25)	3.45 (0.29)	3 (0.50)	3.45 (0.42)	3.50 (1)	3.55 (0.43)
Trunk—Step 10 (6)	2 (1)	2.42 (0.81)	2 (1)	2.41 (0.95)	2 (1)	2.45 (0.76)	2 (1)	2.49 (0.75)

## Data Availability

The dataset is available on request from the authors.

## Supplementary Materials

The following are available online at https://www.mdpi.com/article/10.3390/bioengineering11040400/s1, Figure S1: The Rapid Upper Limb Assessment (RULA) worksheet.
