# Supplementary material for: Ergonomic Analysis of Dental Work in Different Oral Quadrants: A Motion Capture Preliminary Study among Endodontists
Source: Bioengineering (Basel). 2024 Apr 19;11(4):400. doi: 10.3390/bioengineering11040400 (PMC11048138; doi:10.3390/bioengineering11040400)
Supplement: Supplementary file 1 [file bioengineering-11-00400-s001.zip › bioengineering-2892901-supplementary.pdf]

Rapid Upper Limb Assessment (RULA)

The figure shows a worksheet consisting of 15 steps from which an overall attitude (the so-called RULA score) can be derived.

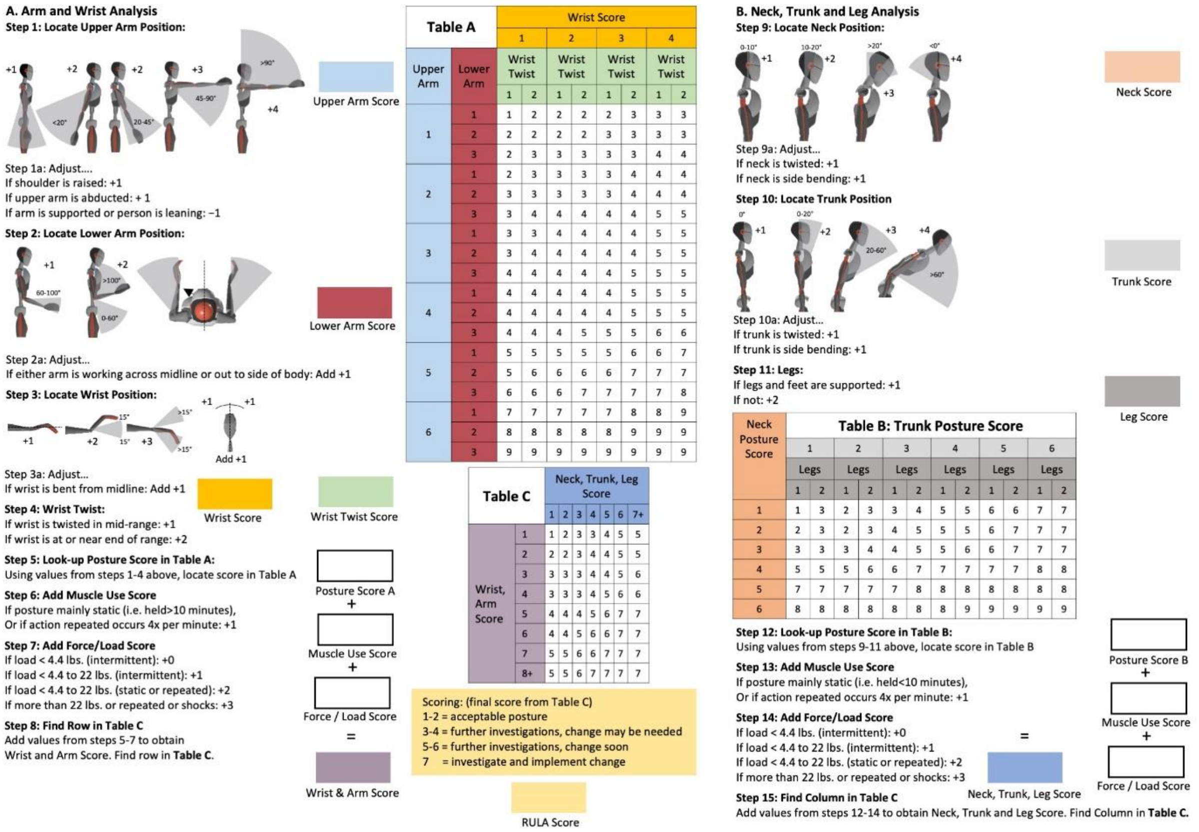

Figure S1. The Rapid Upper Limb Assessment (RULA) worksheet.[49].

How the RULA assessment works, in detail, using the worksheet is described by Maurer-Grubinger et al.[49]

At the end, an overall score is read off a table for the right and left sides of the body; this assesses the ergonomic risk according to the following categorisations[41]:

– Score 1-2: "Posture is acceptable if it is not maintained or repeated for long periods".
– Score 3-4: "Further investigation is needed and changes may be required".
– Score 5-6: "Investigation and changes are required soon".
– Score 7: "Investigation and changes are required immediately".
